# Thermal Behavior Changes of As-Received and Retrieved Bio-Active^®^ (BA) and TriTanium^®^ (TR) Multiforce Nickel–Titanium Orthodontic Archwires

**DOI:** 10.3390/ma16103776

**Published:** 2023-05-17

**Authors:** Angelina Stoyanova-Ivanova, Mirela Georgieva, Valeri Petrov, Jorge N. R. Martins, Laura Andreeva, Alexander Petkov, Nadia Petrova, Velizar Georgiev

**Affiliations:** 1 G. Nadjakov Institute of Solid State Physics, Bulgarian Academy of Sciences, 72 Tzarigradsko Chaussee, 1784 Sofia, Bulgaria; velizar@issp.bas.bg; 2 Faculty of Dental Medicine, Medical University of Sofia, St. G. Sofiiski Blvd., 1431 Sofia, Bulgaria; mirela.georgieva@fdm.mu-sofia.bg (M.G.); v.petrov@fdm.mu-sofia.bg (V.P.); laura_andreeva@fdm.mu-sofia.bg (L.A.); 3 Department of Endodontics, Faculdade de Medicina Dentária, Universidade de Lisboa, Rua Professora Teresa Ambrósio, 1600-277 Lisboa, Portugal; 4 Grupo de Investigação em Bioquimica e Biologia Oral, Unidade de Investigação em Ciências Orais e Biomédicas (UICOB), 1600-277 Lisboa, Portugal; 5 Centro de Estudo de Medicina Dentária Baseada na Evidência (CEMDBE), 1600-277 Lisboa, Portugal; 6 H. H. Wills Physics Laboratory, University of Bristol, Bristol BS8 1TL, UK; al.petkov@bristol.ac.uk; 7 Institute of Mineralogy and Crystallography “Acad. Ivan Kostov”, Bulgarian Academy of Sciences, “Acad. Georgi Bonchev” Str. 107, 1113 Sofia, Bulgaria; nadia5@mail.bg

**Keywords:** austenite finishing temperatures, differential scanning calorimetry method, multiforce Bio-Active^®^ archwires, multiforce TriTanium^®^ archwires, NiTi alloy, orthodontics

## Abstract

Multiforce nickel–titanium (NiTi) orthodontic archwires release progressively increasing forces in a front-to-back direction along their length. The properties of NiTi orthodontic archwires depend on the correlation and characteristics of their microstructural phases (austenite, martensite and the intermediate R-phase). From a clinical and manufacturing point of view, the determination of the austenite finish (Af) temperature is of the greatest importance, as in the austenitic phase, the alloy is most stable and exhibits the final workable form. The main purpose of using multiforce orthodontic archwires is to decrease the intensity of the applied forces to the teeth with a small root surface area, such as the lower central incisors, and also provide forces high enough to move the molars. With the optimally dosed forces of multiforce orthodontic archwires in the frontal, premolar and molar segments, the feeling of pain can be reduced. This will contribute to the greater cooperation of the patient, which is of utmost importance to achieve optimal results. The aim of this research was to determine the Af temperature at each segment of as-received and retrieved Bio-Active^®^ and TriTanium^®^ archwires with dimensions of 0.016 × 0.022 inches, investigated by the differential scanning calorimetry (DSC) method. A classical Kruskal–Wallis one-way ANOVA test and multi-variance comparison based on the ANOVA test statistic using the Bonferroni corrected Mann–Whitney test for multiple comparisons were used. The incisor, premolar and molar segments have different Af temperatures, and they decrease from the anterior to posterior so that the posterior segment has the lowest Af. Bio-Active^®^ and TriTanium^®^ with dimensions of 0.016 × 0.022 inches can be used as first leveling archwires by additional cooling and are not recommended for use on patients with mouth breathing.

## 1. Introduction

In the early 1970s, nickel–titanium (NiTi) alloy was first used for medical applications thanks to its properties of high corrosion resistance, biocompatibility and inherent ability to osseointegrate [1]. Ever since then, NiTi alloy has been increasingly used in orthodontic practice to produce orthodontic archwires, which can be considered one of the main materials in orthodontics as they deliver a low and constant force over long activations [2]. This is a result of its two unique properties: superelasticity and shape memory effects [3,4]. These properties make the NiTi alloy the most popular shape memory alloy in the field of biomedicine [5]. Over the years, the improvement of nickel–titanium (NiTi) archwires has been one of the main goals of manufacturers, with the enhancement of the manufacturing process involving the use of new thermomechanical treatments [6,7]. Heat treatment of NiTi alloy has been frequently used to optimize its microstructure and transformation behavior, increasing its shaping memory characteristics [8,9] and, consequently, improving its mechanical properties [10,11].

All metal alloys used in orthodontics are crystalline, consisting of very specific atom arrangements. Nickel–titanium (NiTi) alloys are characterized by two main types of crystal lattices: a body-centered cubic crystallographic structure named austenite and a more complex monoclinic crystallographic structure named martensite. In nature, there are only 14 possible arrangements, referred to as the Bravais lattices. The different lattice arrangements of an alloy can also be referred to as phases. The mechanical and thermal properties of orthodontic archwires are dependent on both their composition and the phases that are present. The phase that is stable at higher temperatures without stress is the austenitic phase. The initial shape of the archwires (ideal shape) is set when the material is in the austenite phase, and the teeth are moved according to that ideal shape. At low temperatures, a martensitic phase occurs. During the cooling process, the archwires pass from a structure with higher symmetry of the crystal lattice—the austenite one—to the one with lower symmetry—the martensitic one—and, thus, they can remember their original shape (i.e., they have a memory). The NiTi alloy characteristics of “shape memory” and “superelasticity” are exactly due to these crystallographic arrangements [12]. The transition between these two phases/arrangements is fully reversible, occurs at low temperatures and takes place rapidly over some temperature transformation range (TTR). An intermediate phase, termed the R-phase, displaying a rhombohedral atomic arrangement, can form during the forward (heating) transformation from martensite to austenite and the reverse (cooling) transformation from austenite to martensite [13], which are responsible for the two previously mentioned clinically significant properties of NiTi: shape memory and superelasticity [14]. Additionally, the orthodontic archwires, made from NiTi alloy, have a specific transition temperature range (TTR) in which the phase transformation occurs. One of the most useful methods to evaluate the TTR of orthodontic archwires is DSC because it allows the identification of the phase transition temperature and quantity of energy released or absorbed during the heating or cooling processes [15,16,17]. These studies relate to how changes in temperature affect the thermomechanical properties of NiTi alloy, specifically the phase transformation temperatures [18,19,20,21,22,23].

For NiTi archwires with austenite finish (Af) temperatures set much lower than the oral temperature, no “shape memory effect” was observed, but rather superelasticity was observed, as no thermal transition between the two phases occurred [24]. In these cases, at room temperature, the wires are already austenitic. The austenite-martensite phase transition can also be driven by the temperature, so in cases with misaligned teeth, for easier insertion of the archwires in the bracket’s slots, cooling is recommended. Shape memory alloys (martensitic-active) have Af temperatures set close to, or equal to, the oral cavity temperature. The temperature-induced martensite archwires, placed in the mouth at a higher intraoral temperature with induced stress by an applied activation force, will gradually transform to austenite, resulting in the recovery of the ideal preset shape, and, at the same time, the magnitude of the released force will increase [25]. By using a specific manufacturing process, the Af can be set to be close to the intraoral temperature or room temperature [26]. In summary, the final austenite temperature (Af) is a critical factor in the behavior of the archwires, and the temperatures of the phase transformation on each archwire can give a more detailed overview of their physical properties and their in vivo use and applicability [27]. Therefore, it is imperative to select an alloy with an appropriate Af for the clinical case (i.e., less than 37 °C) [25].

Rodrigues et al. showed in a study that the S03 (molar) segment of the studied NiTi archwire (BioForce Dentsply GAC International, Inc., Central Islip, NY, USA) showed the previously mentioned characteristics. The frontal section of the BioForce GAC seems to demonstrate an Af close to the intraoral temperature. This means that this section has a shape memory effect and is more elastic at room temperature, so it can easily be inserted into highly rotated teeth. As the temperature increases, the archwire hardens, transforms into austenite and recovers its shape [28].

A recent study conducted by Roulias et al. [29] aimed to compare the mechanical and thermal properties of the anterior and posterior segments of unused and retrieved commercially available multizone superelastic Bioforce NiTi archwires. The results showed that the Af temperature is higher than regular intraoral temperature, and, even at 2 mm deflections, the forces released are higher than biologically safe limits, which presents a concern.

Orthodontic treatments involve the movement of teeth in various directions and orientations at different stages. These stages are divided into alignment and leveling, correction of the molar relationship and space closure, and finishing treatment. For the alignment and leveling stage, the orthodontist starts with a round light wire that can be bent easily and subsequently replaces it with a thicker wire with a rectangular shape [30,31,32].

Multiforce orthodontic archwires have unique qualities that distinguish them from other types of orthodontic archwires (also made of nickel–titanium alloy) [33]. To this day, they are the only wires that have been developed to release forces with different magnitudes in particular sections of the archwires. This fact is useful for orthodontists, as it shortens the treatment time and reduces the number of visits by patients to the clinics and the number of used archwires during the treatment period. All these facts increase interest in this new type of wire, as it saves clinical time and money.

With our research, another important question for orthodontists is being answered: how long can an archwire stay in the patient’s mouth without changing its most important property—thermal transformation? Popularizing the advantages and the treatment options of these archwires to orthodontic society is a very special task of the study.

Building upon our previous work [34], in this study, we decided to test the possibility of starting the treatment with rectangular cross-section archwires, which possess a specific thermal behavior (0.016 × 0.022 inches). The aim was to determine the austenite finish temperatures at each segment (frontal, premolar and molar) of clinically used Bio-Active^®^ and TriTanium^®^ archwires (up to 8 weeks and over 8 weeks) since the phase transformation temperatures for each orthodontic archwire can provide a more comprehensive understanding and analysis of the physical properties of the archwires and their behavior in clinical use.

## 2. Materials and Methods

### 2.1. Ethics Statement

The clinical procedures were carried out according to the guidelines of the World Medical Association’s Declaration of Helsinki and the Ministry of Health for Good Clinical Practice. Informed consent for testing the archwires was obtained from the patients.

### 2.2. Materials

#### Selection of the Investigated Archwires

Two types of multiforce orthodontic archwires were studied: TriTanium^®^ (3t) archwires (American Orthodontics, Sheboygan, WI, USA) [35] and Bio-Active^®^ archwires (TOMY Inc., Tokyo, Japan) [36] with dimensions of 0.016 × 0.022 inches and a rectangular cross-section. A total of 36 Bio-Active^®^ (TOMY Inc., Tokyo, Japan) (BA) and 36 TriTanium^®^ (3t) (American Orthodontics, Sheboygan, WI, USA) (TR) were explored by the DSC method.

The wires were cut into 15 mm pieces, which correspond to the size of the segments in dental arches. The front segment of the archwire thus coincides with the frontal segment of the dental arch (incisors), and the premolar and molar segments of the archwires correspond to the premolars and molars in the mouth.

The inclusion criteria for this research were archwires taken from patients (a) with permanent dentition; (b) who were treated without extractions; (c) with self-ligating braces; and (d) who were starting treatment with a rectangular cross-section archwire. The exclusion criteria were patients (a) with mixed dentition; (b) those that were treated with extractions; (c) those with conventional types of brackets; and (d) those who were starting treatment with round cross-section archwires.

The pieces were classified into three groups according to the period of clinical use, as follows: I group—as-received (G0) (control group), II group—clinically retrieved up to 8 weeks (G1), III group—clinically retrieved over 8 weeks (G2).

We chose those particular periods of stay in the oral cavity (up to 8 weeks and more than 8 weeks) because the most common time for correction of the initial crowding of the teeth is about 6–8 weeks. In some cases, it takes longer to align the teeth (over 8 weeks). So, we tracked the thermal properties of the used archwires in our study over a longer period to explore the main properties and their eventual changes.

### 2.3. Methods

#### 2.3.1. Disinfection Protocol

Clinically used archwires were taken from patients with fixed appliances (braces) and selected according to the treatment plan. The following disinfection measures were applied: oxygen water 3%, alcohol 95% and final spraying with Isorapid^®^ (Oro Clean Chemie—OCC, Fehraltorf, Switzerland) spray for 1 min. Finally, the samples were washed under running water to remove the disinfectant solutions. After the disinfection protocol, we proceed to segmentation. Using millimeter paper, the exact millimeters were marked on the examined archwires. After marking the areas, the marked places were cut using an orthodontic cutter. The ends of each segment were smoothed with a diamond bur.

#### 2.3.2. Differential Scanning Calorimetry

The differential scanning calorimetry (DSC) method was used to investigate the thermal behavior of the orthodontic archwires, particularly the transition temperature of the phase transformations during their heating and cooling. The samples from different segments of the archwires were studied with a DSC250 (TA Instruments, New Castle, DE, USA). Aluminum crucibles with caps were used, and 4 mg of the material was placed in one of the crucibles, while the other one remained empty to serve as a reference. A calibration was performed using indium as a standard before testing the samples. The temperature accuracy of the device was ±0.05 °C.

**Figure 1:**
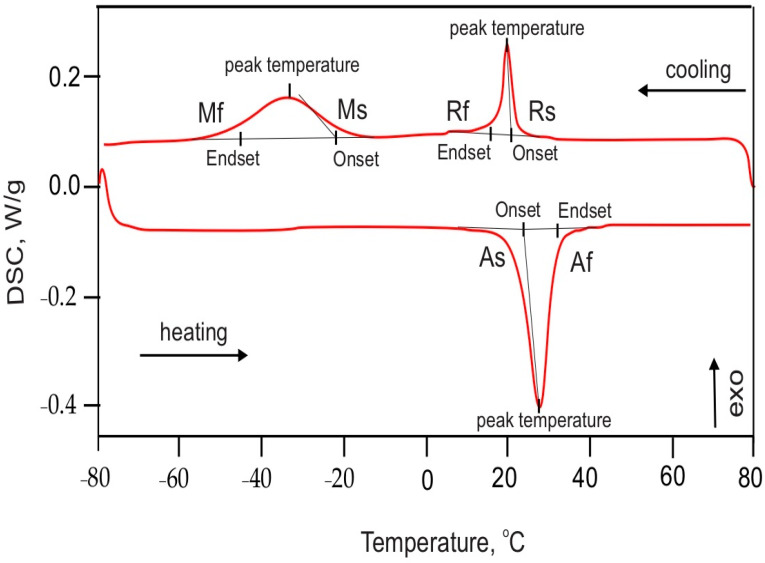
Example of a DSC thermogram of a NiTi orthodontic archwire segment.

The system was heated up to +80 °C and then cooled down to −80 °C at a speed of 10° per minute. An isometric mode was used for 10 min before and after each of the modes. In the heating/cooling chamber, an inert argon gas was streamed with a flow of about 30 mL/min to avoid condensation of the water vapor and acidification of the material. When the sample undergoes a transition related to a change in the enthalpy, this reaction is recorded as endothermic or exothermic. Especially informative is the starting and ending temperature of the separate phases. A DSC thermogram of a sample of a NiTi archwire segment in two modes—heating and cooling—is shown in Figure 1. The effects of the corresponding phase can be clearly observed. When heating up, the starting temperature (As) shows the temperature at which martensite begins to transform into austenite. Af is the temperature at which the austenite transformation finishes. The transition temperatures of these phases, namely starting austenite (As), finish austenite (Af), starting rhombohedral (Rs), finish rhombohedral (Rf), starting martensite (Ms) and finish martensite (Mf), were determined by specialized software (TA TRIOS; TA Instruments, New Castle, DE, USA). The starting and finish temperature of each phase transition were determined from lines tangent to the DSC curve, in which there was a deviation of the adjacent baselines.

#### 2.3.3. Statistical Analysis

The received data from the DSC method were statistically analyzed for the significance of variance between the Af values in the different sections of the archwires (the first part of the statistical analysis). The values of the Af were determined in the different sections of unused archwires as a control group. The second part of the analysis tracked the changes in the Af over the duration of the use of the two archwire types separately for each section. The data gained was statistically analyzed through a Kruskal–Wallis one-way ANOVA test and a multi-variance Mann–Whitney comparison. The validity of the data assumptions in these tests was accomplished using a Shapiro–Wilk normality test and Levene’s homogeneity of variance test. The results were considered statistically significant if they reached a confidence level higher than 95% (*p* < 0.05). This study’s statistical data processing and method implementation were performed in MATLAB^®^ (R2020a) (MathWorks Inc., Natick, MA, USA) through custom and system MATLAB^®^ functions.

## 3. Results

The DSC data for both the Bio-Active^®^ and TriTanium^®^ archwires include data from as-received archwires (before clinical use), as well as clinically retrieved archwires (up to 8 weeks and over 8 weeks of use). The data were collected from two samples, each from both Bio-Active^®^ and TriTanium^®^ archwires, before clinical use. For both the Bio-Active^®^ and TriTanium^®^ archwires retrieved up to 8 weeks, data were collected from five samples each. Similarly, five samples each were used to acquire the data for clinically retrieved archwires used for more than 8 weeks. For the purpose of the analysis, each sample was segmented into frontal, premolar and molar sections, with the Af temperatures measured individually for each segment.

The values of the Af temperatures obtained for the Bio-Active^®^ archwires are given in Table 1 (12 samples in total), while such data for the TriTanium^®^ archwires are given in Table 2 (12 samples in total).

**Figure 2:**
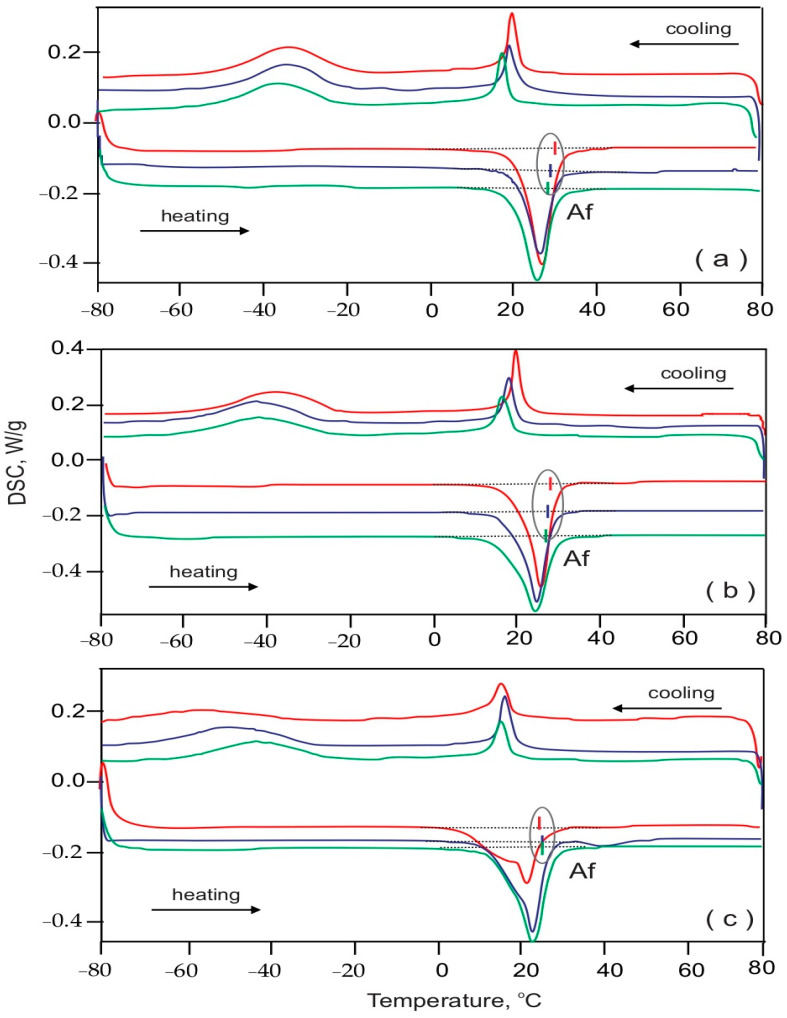
Thermograms of Bio-Active^®^ archwire segments: (**a**) frontal, (**b**) premolar and (**c**) molar. In all graphics, Group I (as-received) archwires are in red color, Group II (retrieved up to 8 weeks) are in blue and Group III (retrieved over 8 weeks) are in green color.

**Figure 3:**
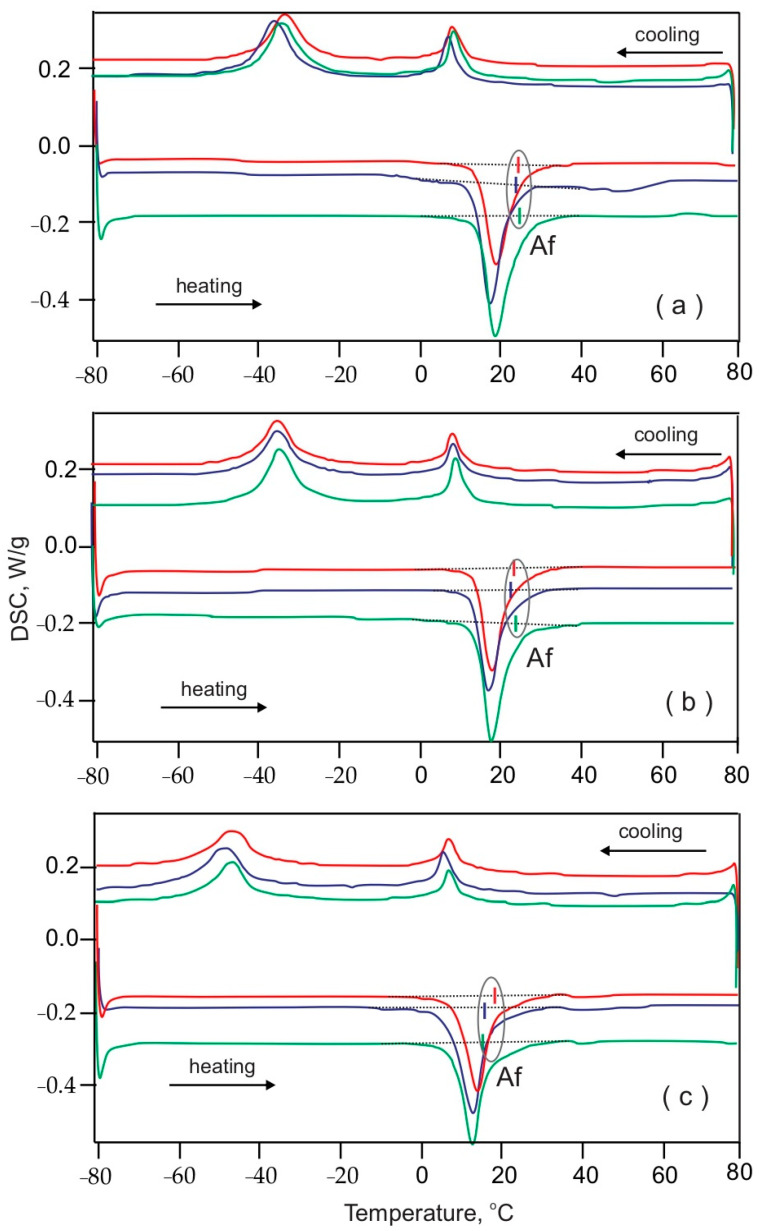
Thermograms of TriTanium^®^ archwire segments: (**a**) frontal, (**b**) premolar and (**c**) molar. In all graphics, Group I (as-received) archwires are in red color, Group II (retrieved up to 8 weeks) are in blue and Group III (retrieved over 8 weeks) are in green color.

Figure 2 and Figure 3 present the DSC curves of the representative pre-selected samples from the general three segments (frontal, premolar, molar) in the three periods of use: Group I as-received (red lines), Group II retrieved up to 8 weeks (blue lines) and Group III retrieved more than 8 weeks (green lines) of both the Bio-Active^®^ and TriTanium^®^ archwires. Following the Af dislocation with respect to the temperature, the subsequent observations were made: (i) generally, the Af values were lower for the TriTanium^®^ archwires compared to those of the Bio-Active^®^ archwires; and (ii) for both the Bio-Active^®^ and TriTanium^®^ archwires, the tendency for different Af temperatures in the frontal, premolar and molar segments was kept during the whole period of clinical use.

An analysis of the statistical significance of variation within the datasets was carried out using the classical one-way ANOVA test. The significance level of the differences between the different groups in the data was determined using Bonferroni corrected Mann–Whitney multiple comparative tests (MCT) based on the full ANOVA statistics. This test works under the assumption that the datasets have normal population distribution, as well as the same variance. The Shapiro–Wilk normality test was used to check the validity of the first assumption, while Levene’s homogeneity of variance test was used to check the validity of the latter. Both tests returned a *p*-value of <0.001, which suggests no significant deviation from these initial assumptions.

For the purpose of the ANOVA and MCT tests in the analysis, a confidence level of over 95% (*p* < 0.05) was considered a statistically significant result. The statistical data processing in this work was carried out through system and custom functions in MATLAB. Carrying out this analysis allowed for the identification of statistically significant differences in the Af temperature depending on the archwire type (Bio-Active^®^ or TriTanium^®^) as well as the basis of the segment (frontal, premolar or molar) and time of use (unused, up to and exceeding 8 weeks).

The obtained data are presented below in bar plot figures based on the ANOVA statistics. The bars represent the mean value of the compared quantity (Af in this case) in each group. The error bars in red show the standard deviation of the mean value. The plots also mark the significant differences between the mean values for the presented groups. A “significant difference” here implies a difference that was likely caused by something other than random or sampling error. The horizontal lines represent a statistically significant variance between a pair of groups in the data, marked with asterisks depending on the confidence level (* for *p* < 0.05; ** for *p* < 0.01; *** for *p* < 0.001).

**Figure 4:**
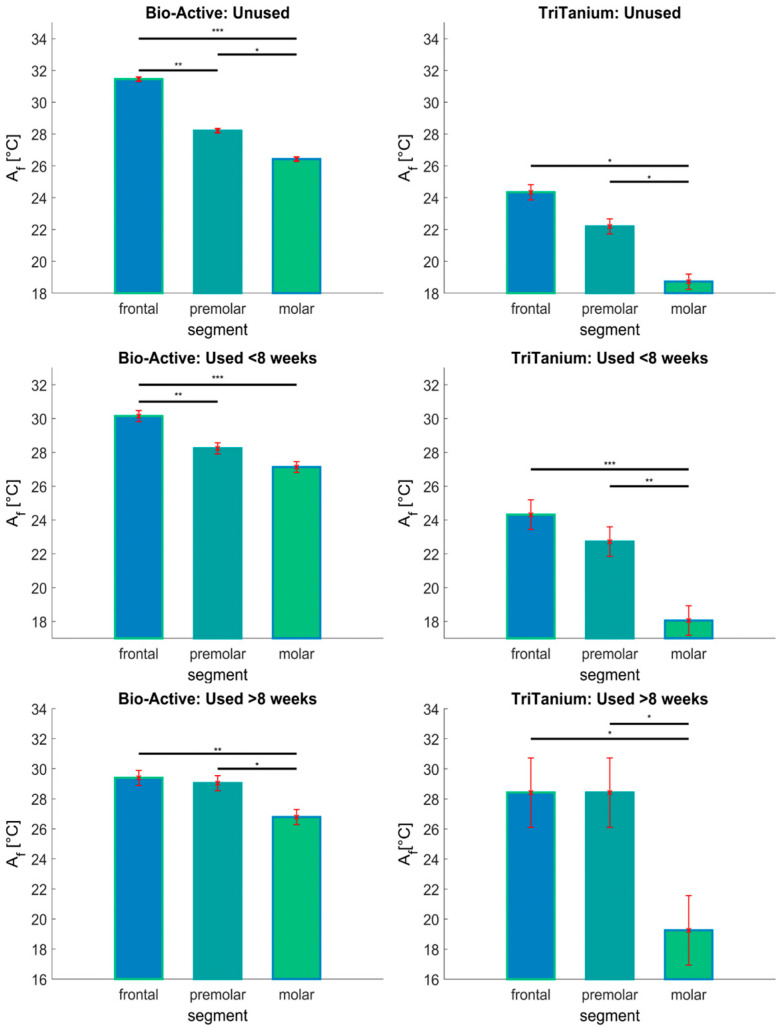
Austenite finish temperatures for Bio-Active^®^ and TriTanium^®^ archwires, unused (as-received), used less than 8 weeks and used over 8 weeks (retrieved). Data are grouped by the archwire segments (* *p* < 0.05; ** *p* < 0.01; *** *p* < 0.001).

**Figure 5:**
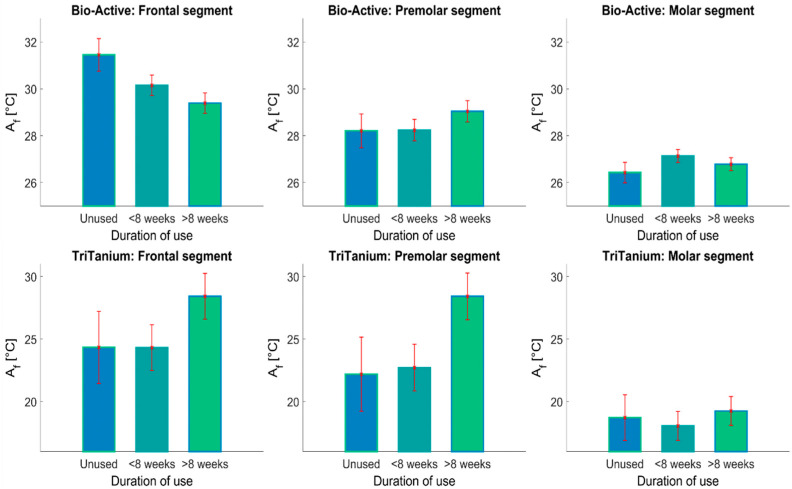
Austenite finish temperatures for Bio-Active^®^ and TriTanium^®^ archwires in the frontal, premolar and molar segments. Data are grouped by duration of use of the archwires.

Based on ANOVA statistics, one may produce a mean bar plot with error bars Figure 4 and Figure 5. The X-axis shows the compared groups. The bars represent the mean of the compared quantification measure for each group (Y-axis). The group mean is represented by a bar. Error bars are lines from the top of the bar parallel to the Y-axis, representing the uncertainty or SD of the mean value and the confidence intervals are vertical red lines. If there is a black horizontal line above 2 bar-s, then the difference between the means of the two compared groups is statistically significant. In addition, the *p*-value is marked with asterisks—(* *p* < 0.05; ** *p* < 0.01; *** *p* < 0.001).

As seen in the first row in Figure 4, the data for the as-received archwires showed a significant difference in the Af depending on the segment (frontal, premolar or molar), as well as between the two types of archwires—Bio-Active^®^ and TriTanium^®^. The measured Af values for the as-received Bio-Active^®^ archwires varied between 31 °C and 26 °C for the frontal and molar segments, respectively, while the same variation in the case of the TriTanium^®^ archwires was between about 24 °C and 19 °C. One can appreciate from Figure 4, that the Af has a consistently lower mean value in the TriTanium^®^ archwires compared to the Bio-Active^®^ archwires in each considered case (taking into account both the different segments and durations of use).

This was also reflected in the MCT test, where the significance in the data variation between the Bio-Active^®^ and TriTanium^®^ archwires was determined with a confidence level of *p* < 0.001. There was also significant variance in the Af temperature values for each archwire type depending on the segment, with a general trend of the phase transition temperature decreasing monotonically from the frontal towards the molar segments. This is similarly reflected in the MCT test, where the significance of the variance in the Af depending on the archwire segment was determined with a confidence level of *p* < 0.001. This means that the difference in the phase transition temperatures in the Bio-Active^®^ and TriTanium^®^ archwires, depending on the segment, was statistically significant. Our multi-comparison test analysis also highlights the statistically significant variance between the data for the TriTanium^®^ molar segments compared to the frontal and premolar ones, regardless of the duration of use. The Bonferroni *p*-value for the comparison between the molar and both of the other segment types in the TriTanium^®^ archwires was obtained as <0.001.

On the other hand, the general variance in the data, depending on the duration of use, for the archwires was not determined to be statistically significant (with a *p*-value of 0.454). Figure 5 shows a breakdown of the data grouped by the duration of use to help illustrate this point. For the frontal segments of the Bio-Active^®^ archwires, it was possible to see a significant decrease in the Af temperature between the as-received archwires and the ones retrieved up to 8 weeks. There was, however, no statistically significant change in the Af value between the frontal segments used for less than 8 weeks and those used for longer periods of time.

For the premolar segments of the Bio-Active^®^ archwires, a slight trend of increasing Af temperature with increased duration of use was observed. This increase, however, was not statistically significant (*p*-value of 0.36). Similarly, no statistically significant differences in the Af were observed for the Bio-Active^®^ molar segments. There was also no statistically significant variance in the phase transition temperatures in any of the segments of the TriTanium^®^ archwires due to the duration of use (*p*-value of 0.254). While the apparent increase in the Af temperature in the frontal and premolar segments of the TriTanium^®^ archwires after more than 8 weeks of use in Figure 5 looked notable at a glance, this may be due to outliers in the data. Thus, the apparent inflation of the mean in those groups was still not of statistical significance.

## 4. Discussion

In the present study, conducted on orthodontic archwires with differentiated force release— Bio-Active^®^ and TriTanium^®^—using the DSC method, it was observed that these archwires have different Af temperatures in their frontal, premolar and molar segments (Table 1 and Table 2). Statistical analysis of Group I (as-received archwires) showed that the frontal segments of both types of wires had the highest Af temperature, but the temperature was higher in the Bio-Active^®^ archwires than in the TriTanium^®^ ones (31.5 °С > 24.5 °С). Statistically significant differences in the Af temperatures were observed in the premolar and molar segments of the Bio-Active^®^ archwires.

The TriTanium^®^ archwires in Group I showed different phase transformation temperatures in all three segments. However, no statistically significant differences were found between the frontal and premolar segments. Statistical differences were noted between the frontal and molar segments. The premolar and molar segments of the TriTanium^®^ archwires had Afs of 22 °С and 19 °С, respectively. In comparing the premolar and molar segments of the TriTanium^®^ archwires with the Bio-Active^®^ archwires, the TriTanium^®^ wires had much lower phase transition temperatures.

In our present research, no statistically significant differences were reported in the Af in the different segments of the Bio-Active^®^ and TriTanium^®^ archwires during the periods of use. The Af remained unchanged in Group I, Group II and Group III.

The phase transition temperatures for the TriTanium^®^ archwires were below the temperature of the oral cavity and closer to room temperature. That means that at room temperature, before being inserted into the bracket slots, it is already in an austenite phase. TriTanium^®^ belongs to the group of austenitic active wires and possesses superelasticity.

The mechanical properties are in direct relation with the thermal behavior of the archwires and their Af temperatures, which are set during their production process. In a previous study, it was established that the studied orthodontic archwires (Bio-Active^®^ and TriTanium^®^) were multiforce and could release varying forces along their length. The weakest forces were observed in the frontal segment, the medium forces were observed in the premolar segment, and the strongest forces were noted in the molar segment [33]. The frontal segments of both types of archwires possess the highest Af temperatures and release the weakest forces in comparison to the premolar and molar segments. At 2 mm deflection, the mean released forces of the Bio-Active^®^ archwires for the frontal segments are between 3.8 N and 4 N. For the premolar segment, the measured mean forces are between 4 N and 4.2 N, and the molar segment releases a force value of 5 N. At a 2 mm deviation of the TriTanium^®^ archwires, the frontal segment shows a force release of 4 N; the premolar segment shows a force of 4.5 N; and the molar segment shows a force between 5 N and 6 N. In the present study, it was observed that the TriTanium^®^ archwires released higher forces than the Bio-Active^®^ archwires. By connecting the previous and current studies, one may suggest that the lower the set Af temperature is, the higher the forces released by the archwires. In comparing the force to the Af temperatures in the segments for the Bio-Active^®^ as-received archwires, the following values were observed: frontal segment 31 °C/3.8 N, premolar segment 28 °C/4 N and molar segment 26 °C/5 N. In comparing the force to the Af temperatures in the segments for the TriTanium^®^ as-received archwires, the following values were observed: frontal segment mean 24 °C/4 N, premolar segment mean 22 °C/4.5 N and molar segment mean 18 °C/between 5 N and 6 N [33]. The Bio-Active^®^ archwires can be classified into the group of martensitic-active wires (heat-activated wires without copper) because the manufacturers set the Af to be above room temperature and close to the temperature of the oral cavity. Thus, at room temperature, they are still flexible and are easily engaged into rotated teeth. As the temperature increases, a thermal transition from martensite to austenite takes place, and the so-called “shape memory” effect happens. The frontal segment of the dental arch (incisive segment) is characterized as having more misaligned teeth than premolar and molar segments. The frontal segment of the Bio-Active^®^ archwires with the Af temperature set close to the oral temperature (31 °C) is at room temperature in a phase between austenite and martensite so the archwire is malleable and elastic. The transformation from martensite to austenite happens in the mouth when the temperature rises above 31 °C and the archwire hardens. This thermal behavior allows the orthodontist to engage archwires with a bigger size and rectangular cross-section in misaligned teeth. The results confirm that the studied Bio-Active^®^ and TriTanium^®^ archwires are thermodynamic, and, thus, at higher room temperatures, pre-cooling with a spray is recommended to induce a thermal transition between the phases.

In a study conducted by Ohara, the frontal segment of BioForce GAC archwires showed an Af close to that of the oral cavity, while the back segment showed an Af temperature close to room temperature [24]. A recent study detected statistically significant differences in the As and Af transformation temperatures between the anterior and posterior segments of BioForce archwires in new and retrieved specimens. However, aging did not significantly affect the transformation temperatures [29].

The differences in the phase transformations between unused and used CuNiTi archwires were evaluated after several weeks of clinical use. The authors determined that there were no significant differences in thermal activity between unused and used archwires. In thermoactive archwires with a transition temperature of +27 °C, set by the manufacturer, a significant reduction in the enthalpy was detected [37,38].

A Japanese collective thermomechanically studied three types of archwires with a size of 0.016 × 0.022 inches (martensite-active with a shape memory effect, austenite-active with superelasticity and thermodynamic NiTi orthodontic archwires). Using DSC analysis at temperatures of 0 °C, 20 °C, 37 °C and 60 °C, they found that statistically significant differences were present in both the phase transition and Af temperatures. The Af of the martensite-active wires was 52.4 °C, of the austenite-active wires was 19.7 °C and of the thermodynamic wires was 24.6 °C [39].

Thermoactivated orthodontic archwires transform into martensite almost completely during clinical use at an Af temperature of over 37 °С and are elastic extra-oral as well as intraoral. The forces applied to the teeth are remarkably low, and, therefore, such orthodontic archwires are recommended for treating patients with periodontal problems. Austenite forms only when the environment temperature exceeds 40 °C.

Consuming hot drinks is considered to increase the released forces, which speeds up teeth movement. Cold drinks will decrease the forces. The dynamics of oral cavity temperature over a 24 h period have been studied in several ways, but the mean temperature reported is about 35 °С, with differences occurring over time and at different locations in the mouth [40,41]. The temperature range is generally wide, up to 50 °С, although peak values were reached only for very short periods and mostly in the palatal area. Most of the time (79%) the mouth temperature ranges from 33 °С to 37 °С. For these types of archwires, Sakima et al. concluded that at 30 °C there is no plateau of deactivation, which means that at that temperature the archwires do not transition into austenite [26].

The conventional wire progression—stepping through multiple rounds of progressively greater size and force—results in forces that overpower some teeth and underpower others. The value of this new wire technology lies in its capacity to apply physiologically appropriate forces to each individual tooth, thereby minimizing the formation of avascular necrotic tissue and shortening the lag phase [32]. The advantages of shape memory archwires are that they release weaker forces at the same dimensions (sizes) when compared to other non-elastic types of archwires. So, for clinicians, it is possible to start with bigger-sized archwires, Bio-Active^®^ and TriTanium^®^, as the first levelling wire. In addition, the patient can control the level of pain by drinking hot or cold drinks. A major disadvantage is that, in this way, the released forces cannot be calculated and the control of the teeth movement is harder [42].

The limitations of the present study are that the forces can vary according to environmental conditions and can be difficult to accurately predict. Thus, a possibility for a future study is to explore ways to exercise precise control over the external factors that influence the mechanical and thermal properties of the archwires and further study their relationship. In addition, lately, there have been studies on the effects of the fluoride content of mouthwashes on the superelastic properties of NiTi archwires [43]. This is of potential interest for future studies on the properties of the archwires that have been studied in this research.

## 5. Conclusions

In summary, the main advantages of the studied multiforce Bio-Active^®^ and TriTanium^®^ archwires are as follows:

- Bio-Active^®^ archwires can be classified into the group of martensite-active wires (heat-activated) due to the fact that the manufacturers set the Af to be above room temperature and close to the temperature of the oral cavity.
- The Af temperatures of TriTanium^®^ archwires, classified as austenite-active (superelastic) archwires, are set quite below the temperature of the oral cavity. This means that at room temperature, before engaging the archwires into the bracket slot, they are already in the austenitic phase.
- The investigated as-received Bio-Active^®^ and TriTanium^®^ orthodontic archwires had different Af temperatures in the three segments. The frontal segments possess the highest Af, followed by the premolar segments. The lowest Af temperatures were reported for the molar segments.
- In general, the Af was lower in all the segments in the TriTanium^®^ archwires when compared to the Bio-Active^®^ archwires, both the unused ones as well as the ones used up to and over 8 weeks.
- The thermal behavior does not change after clinical exposure.
- Bio-Active^®^ and TriTanium^®^ archwires with dimensions of 0.016 × 0.022 inches can be used as first leveling archwires by additional cooling and are not recommended for use on patients with mouth breathing.
- The thermal properties of the studied thermodynamic multiforce archwires depend on external factors, such as the environmental temperature, sleeping and/or staying with the mouth open and eating and/or drinking cold foods and beverages.

## Figures and Tables

**Table 1:** DSC data from Bio-Active^®^ archwires.

Af Temperatures from Bio-Active^®^ Archwires [°C]								
Before Clinical Use			Used up to 8 Weeks			Used over 8 Weeks		
Frontal	Premolar	Molar	Frontal	Premolar	Molar	Frontal	Premolar	Molar
31.45	28.21	26.17	30.39	28.10	27.36	30.23	28.79	27.45
31.45	28.21	26.69	30.32	28.48	27.42	26.90	27.48	26.46
			29.99	28.84	27.69	30.36	30.28	27.70
			29.99	26.39	26.50	29.28	29.15	26.37
			30.06	29.38	26.70	30.18	29.49	25.95

**Table 2:** DSC data from TriTanium^®^ archwires.

Af Temperatures from TriTanium^®^ Archwires [°C]								
Before Clinical Use			Used up to 8 Weeks			Used over 8 Weeks		
Frontal	Premolar	Molar	Frontal	Premolar	Molar	Frontal	Premolar	Molar
23.65	21.85	19.02	23.35	20.45	15.87	30.36	26.88	22.26
25.03	22.53	18.42	22.45	21.39	16.76	25.09	23.84	16.69
			25.17	24.90	17.45	24.63	23.97	17.67
			25.50	24.53	22.15	38.06	37.36	22.79
			25.11	22.33	18.03	23.95	24.99	16.85

## Data Availability

The datasets used and/or analyzed during the current study are available from the authors upon reasonable request.
